# Supplementary material for: Single-neuron dynamical effects of dendritic pruning implicated in aging and neurodegeneration: towards a measure of neuronal reserve
Source: Sci Rep. 2021 Jan 14;11:1309. doi: 10.1038/s41598-020-78815-z (PMC7809359; doi:10.1038/s41598-020-78815-z)
Supplement: Supplementary file 1 — Supplementary Information 1. [file 41598_2020_78815_MOESM1_ESM.pdf]

Single-neuron dynamical effects of dendritic pruning implicated in aging and neurodegeneration: Towards a measure of neuronal reserve

Christoph Kirch[1,2], Leonardo L. Gollo[1,2,3]

[1]QIMR Berghofer Medical Research Institute, Brisbane, Australia

[2]The Queensland University of Technology, Brisbane, Australia

[3]Monash University, Melbourne, Australia

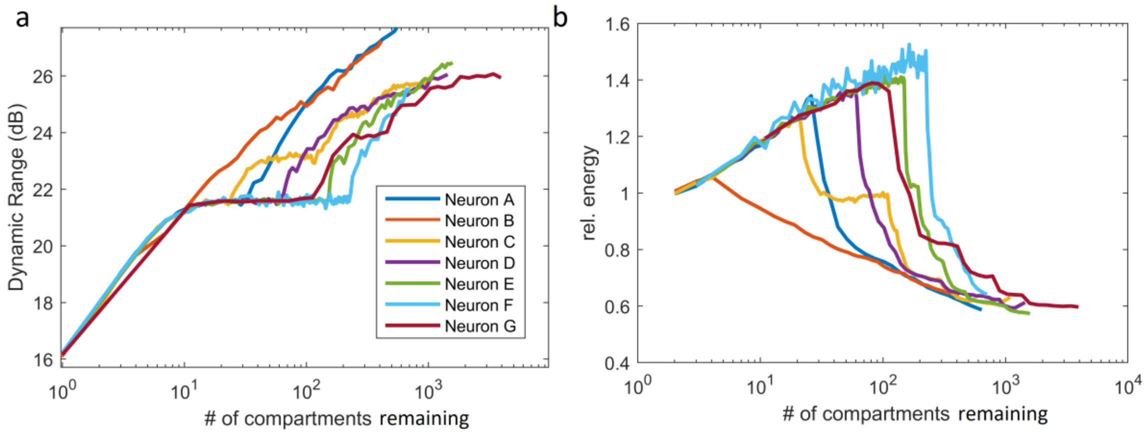

Supplementary Figure 1: Convergence of dynamics with neuronal pruning. The number of compartments reduces as pruning accumulates. (a) Dynamic range versus the number of compartments remaining. (b) Relative energy consumption versus the number of compartments remaining.
